# Scalable Rules for Coherent Group Motion in a Gregarious Vertebrate

**DOI:** 10.1371/journal.pone.0014487

**Published:** 2011-01-05

**Authors:** Marie-Hélène Pillot, Jacques Gautrais, Patrick Arrufat, Iain D. Couzin, Richard Bon, Jean-Louis Deneubourg

**Affiliations:** 1 Université de Toulouse, UPS, Centre de Recherches sur la Cognition Animale, Toulouse, France; 2 CNRS, Centre de Recherches sur la Cognition Animale, Toulouse, France; 3 Service d'Ecologie Sociale, CP 231, Université libre de Bruxelles, Plaine Campus, Brussels, Belgium; 4 Department of Ecology and Evolutionary Biology, Princeton University, Princeton University, Princeton, New Jersey, United States of America; University of Bristol, United Kingdom

## Abstract

Individuals of gregarious species that initiate collective movement require mechanisms of cohesion in order to maintain advantages of group living. One fundamental question in the study of collective movement is what individual rules are employed when making movement decisions. Previous studies have revealed that group movements often depend on social interactions among individual members and specifically that collective decisions to move often follow a quorum-like response. However, these studies either did not quantify the response function at the individual scale (but rather tested hypotheses based on group-level behaviours), or they used a single group size and did not demonstrate which social stimuli influence the individual decision-making process. One challenge in the study of collective movement has been to discriminate between a common response to an external stimulus and the synchronization of behaviours resulting from social interactions. Here we discriminate between these two mechanisms by triggering the departure of one trained Merino sheep (*Ovis aries*) from groups containing one, three, five and seven naïve individuals. Each individual was thus exposed to various combinations of already-departed and non-departed individuals, depending on its rank of departure. To investigate which individual mechanisms are involved in maintaining group cohesion under conditions of leadership, we quantified the temporal dynamic of response at the individual scale. We found that individuals' decisions to move do not follow a quorum response but rather follow a rule based on a double mimetic effect: attraction to already-departed individuals and attraction to non-departed individuals. This rule is shown to be in agreement with an adaptive strategy that is inherently scalable as a function of group size.

## Introduction

Elucidating the mechanisms governing cohesion during group movement is a central issue to our understanding of the evolution of social behaviour [1]. In order to maintain the benefits of group living (such as reduced predation risk, better foraging efficiency and the exchange of social information), mobile animals often have to synchronize their activities, forage collectively and move together by coordinating both the timing and direction of their movement decisions.

Collective movements typically begin with some individuals first departing to a new area. Thus, movement initiations within resting or foraging group are instances of transient group splitting. Decision-making regarding movement may be especially critical for those first individuals that leave the group since they disproportionately increase their risk of predation [2] and potentially lose territorial defense benefits [3]. If benefits are linked to group size, as is expected [4], there must exist some conflict between staying with others and taking the risk of departing to forage on higher quality resources or to reduce competition. Importantly, this conflict between leaving and staying also concerns not only the first individual to initiate the movement (the “initiator” [5]), but also those individuals which have not yet departed. When some of the group members decide to move, the remaining individuals have to choose whether to follow those that have departed. If they do not, the group will remain split.

Although individual movement decisions are known to be influenced by the actions of conspecifics [6]–[8], the precise mechanisms are largely unknown [9]. A way of addressing this is to identify the stimulus-response function at the individual scale, that is the individual following rule that can account for the observed collective outcomes. Many theoretical or experimental studies have suggested that collective decisions to move emerge either from a kind of a pre-departure consensus building based on a voting procedure [1], [10], or from a combination of more individualistic decisions based on a behavioural switch when a quorum has been reached [9], [11]–[16]. In most cases, however, they postulate the decision-making process at the individual scale and then test the model predictions at the collective scale, without explicit reference to experimental data at the individual scale [17]. However, different models at the individual scale can lead to the same predictions at the collective scale, provided their parameters can be freely adjusted. As a consequence, conclusions drawn from such models remain hypothetical regarding the full details of the information used by individuals to come to their decisions.

To gain deeper insight into collective motion in animals, and to highlight the individual decision-making process, we analyzed quantitatively the individual responses in the course of collective departures for different groups of sheep (Merino breed). For this, we trained individual sheep to move towards a panel raised at the periphery of an arena [18]. A single trained individual was then introduced into groups of naïve sheep and used to initiate a collective movement. To identify the nature of the stimuli that trigger individuals' decisions to follow we characterized the stimulus-response function at the individual scale for all naïve individuals. Under our experimental conditions, in which environmental factors are controlled, the stimulus is purely social, and was provided by the behaviour of other group members. A key feature was the use of groups of different sizes (*N* = 2, 4, 6, 8) so that sheep were exposed to various combinations of two factors: the number of departed individuals (including those sheep departing in response to the trained leader) and the number of non-departed individuals. We quantified the individual stimulus-response function by the probability per unit time to depart (or departure rate, expressed in s^−1^) when exposed to such combinations. Both factors (the number of departed and non-departed neighbours) were shown to significantly affect the departure rate.

The insight that individuals integrate information about their departed and non-departed neighbours has several important functional consequences. First, the collective dynamics remain the same in groups of any size, and it therefore supports scalability at least up to group sizes where each individual can see each other. Furthermore, the parameter values that fit experiments are precisely the ones that minimize the duration (and thus potential costs) of the temporal split of the group that is the time elapsed between the trained departure and the departure of the last follower.

## Results

**Figure 1:**
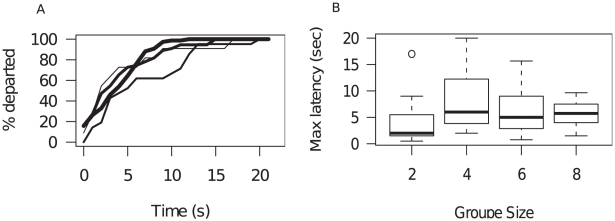
Kinetic of collective departures. A) Average kinetic of collective departures do not depend on group size as expressed as the percentage of departed individuals in function of the time for group of *N* = 2, 4, 6 and 8 individuals (from thin curve to thick curve respectively). B) Time course of collective departures as function of the group size.

In all experiments, a consensus decision was observed. The departure of the trained sheep towards the visual panel always triggered a collective movement and all naïve sheep followed within a relatively short time (95% followed in less than twelve seconds). Moreover, the time course of collective departures did not depend on the group sizes (Kruskal test on time course: *χ^2^* = 2.045, *df* = 3, *P* = 0.56, Fig. 1) whilst one may have expected that larger groups would take a longer time to depart, even over this range of group sizes.

Individuals' behavioural responses were quantified by calculating the departure rate separately for each combination of departed/non-departed group members. To quantify this departure rate, we assumed a continuous time Markovian jump process, that is, the probability per unit time displaying the response (in this study, following) is constant over time as long as the stimulus remains the same, and this probability jumps to a new value when the stimulus changes. This assumption was validated (see Data analysis and Appendix S1). To identify the nature of the stimulus, we considered that the state of the group changed each time a further individual followed. For instance, in a group of four individuals (one trained and three naïves), each naïve individual is assumed to witness the same group state from the time the trained individual departed until the first follower's departure. Then, from this departure until the next, the two still non-departed naïves witness a new group state which consists of the two departed individuals (the trained and the first follower) and one non-departed individual, and so on. Each time the group state changes, the rate of following may or may not change, depending on what the sheep are reactive to. Accordingly, if the rate of following changes from one group state to another, the two states can be considered as different stimuli.

**Figure 2:**
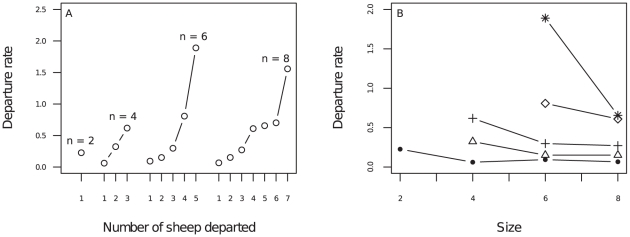
Individual stimulus/response function. Departure rates are plotted (A) as a function of the number of already departed sheep *D* in each group size (*N* = 2, 4, 6 and 8), and (B) for each follower's rank departure as function of the group size (dot: rank 1, triangle: rank 2, cross: rank 3, square: rank 4, star: rank 5). Note that the corresponding number of non-departed sheep is *N*−*D*.

For all group sizes, the departure rate increased sharply with the number of departed individuals: individuals were increasingly stimulated to depart as the number of departed animals increased (Fig. 2A). Moreover, for a given number of departed individuals, the departure rate decreased with the number of non-departed individuals (Fig. 2B). For instance, when three individuals were already departed, the departure rate decreased from 0.62 s^−1^ in groups of four (one non-departed) to 0.30 s^−1^ in groups of six (three non-departed) to 0.27 s^−1^ in groups of eight (five non-departed). This suggests that sheep were responsive both to the departed and non-departed individuals since the following rate changed each time either the number of departed or the number of non-departed individuals changed.

### Models of the individual decision

To verify this hypothesis, we tested the relevance of this model against more parsimonious models: responding only to the trained sheep departure (initiation), or responding only to the departed individuals. For testing, we used one simple equation:
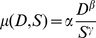
(1)where *μ* is the departure rate (the response), α is the probability to follow when there is only one follower and one departed individual and *D* and *S* the number of departed and non-departed individuals respectively (the stimulus). Note that *μ* is null before the trained departure (*D* = 0), which is consistent with the experiments since the collective movement was triggered by the trained individual in all cases (no naïve individual's departure towards the panel observed before the trained individual's departure). The parameters β and γ modulate the influence of *D* and *S*, and allow testing the three alternative models according to their values,

*Model 1 : Just mimic the initiator*

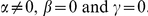
Under model 1, the following decision is stimulated by the departure of the initiator only, and is independent of whether other group members have departed or not. Therefore the departure rate should be the same for all naïves at any time, following 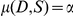.

*Model 2 : Mimic all the departed individuals*

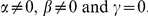
The following decision is stimulated by the initiator and also by the already departed group members. The departure rate should monotonically increase with the number of departed animals, following 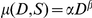.

*Model 3 : Mimicking the departed or staying with non-departed*

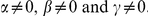
The following decision is stimulated by the group members which have already departed, as in model 2, but is concurrently inhibited by the ones which have not. The departure rate should increase with the number of departed animals, but decrease with the number of non-departed individuals, following equation (1).

To test the adequacy of each model, we first adjusted the corresponding free parameters to the entire set of experimental values (departure rates, Fig. 2A). Note that fitting model 3 required experiments with different group sizes, so that *D* and *S* are not colinear. Both factors had a significant effect in the full model (regression in the log domain: 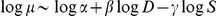, respectively *P*_β_<10^−6^ and *P*_γ_<2.10^−4^, *F*_2,13_ = 102.9, *P*<10^−7^, *r^2^* = 0.94). To test the likelihood that model 3 is a better explanation than model 2 and model 1, we derived their corresponding AIC (Akaike Information Criterion, Table 1) [19]. Model 3 with both factors (departed and non-departed) is orders of magnitude (1400 times) more likely to be the best explanation for following rates compared to model 2 with departed individuals only.

**Table 1:** Model selection with AIC.

Model	Factors	K	RSS	AICc	Δi	Wi
Model3	D, S	4	0.977	−33.096	0	0.999
Model2	D	3	3.032	−18.612	14.483	7.15 10−4
Model1	None	2	16.449	5.366	38.462	4.44 10−9

For each model, the AIC value was computed using bias-adjustment for small sample sizes according to: AICc = n*ln(RSS/n)+2*K+(2*K*(K+1))/(n−K−1), where n is the number of data, RSS the residuals sum of squares and K the number of parameters (25). The plausibility of each model is assessed by its corresponding *Akaike weight* Wi which was obtained by normalizing the relative likelihoods exp (−0.5*Δ**i)**, with Δi the difference between the AICc of the model i and the lowest AICc. The plausibility of model 3 versus model 2 is given by Wi(model 3)/Wi(model 2) = 0.999/7.15 10^−4^ = 1400.

### Models' predictions at the collective scale

**Figure 3:**
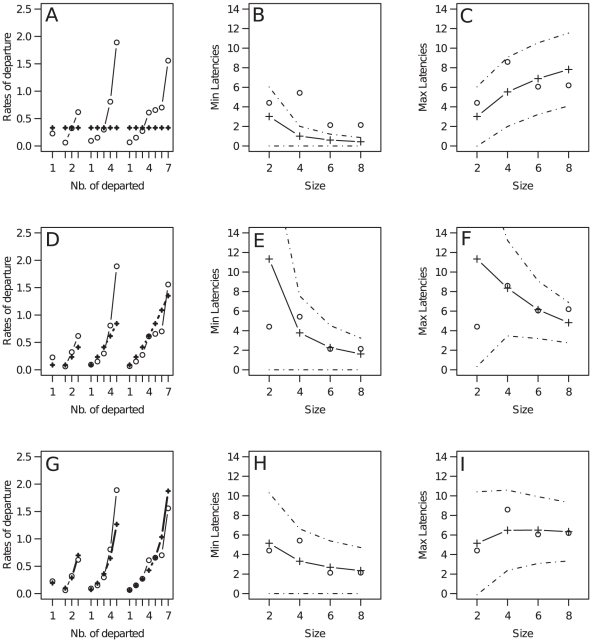
Comparison of models' predictions with experimental data. Comparison of the mean experimental (empty circle) and expected values (cross) obtained under (A–C) model 1 (sheep decision to follow is independent of other sheep), (D–F) model 2 (following is prompted by the number of already departed sheep) and (G–I) model 3 (sheep decision depends on the number of departed and non-departed sheep). Left column represent the individual stimulus / response function as a function of the number of already departed sheep *D* in each group size (*N* = 2, 4, 6 and 8). Note that the corresponding number of non-departed sheep is *N*−*D*. Mid and right columns represent the corresponding results at the collective scale: mean latency of the first follower and mean duration of the move (i.e. mean duration from the trained' departure and the last follower's departure). Dashed lines represent the standard deviation.

We used the departure rates fitted under each model (Fig. 3, left column, cross symbols) as input to compute the corresponding dynamics of the followers' departures. The model predictions obtained at the collective scale were compared to the experimental values for (*a*) the mean latency of the first follower's departure (Fig. 3, middle column) and (*b*) the mean duration from the trained individual's departure to the last follower's (Fig. 3 right column). These predictions were derived from:
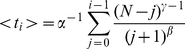
(2)which is the mean latency of the *i* ^th^ follower's departure (see Appendix S2). <*t_1_*> corresponds to the mean latency of the first follower (*a*), and <*t_N_*> corresponds to the mean latency of the last follower, which is the same as the mean duration of the collective move (*b*).

Under model 1 the departure rates are independent from the number of departed and non-departed conspecifics. This model enforces a distribution of the fitted rates greatly different to the data (Fig. 3A, α = 0.33 s^−1^). Accordingly, it yields bad predictions at the collective scale: the predicted first follower's mean latency is far too low while the mean duration increases continuously with an increasing group size (Figure 3B–C).

Model 2 allows a distribution of the fitted rates that is closer to the observed responses since they are allowed to reflect the stimulating effect of the number of departed individuals (Fig. 3D, α = 0.09, β = 1.4, *r^2^* = 0.80). However, the model still yields incorrect predictions for small groups (Fig. 3E–F).

Only model 3, which also includes an inhibiting effect of still non-departed conspecifics, provides an accurate distribution of the fitted rates (Fig. 3G, α = 0.19, β = 1.16 and γ = 0.6, *r^2^* = 0.94, see above). Accordingly, it yields predictions that are consistent with the experimental data at both the individual and collective level (Fig. 3H–I).

This supports the hypothesis that individual response depends both on the number of departed and non-departed animals. Therefore our analysis reveals that sheep decision-making is not based on a single mimetic effect but rather on a rule which balances two mimetic opposite effects: follow the departed individuals but remain with the non-departed individuals. Hence, not only do sheep respond to the sudden events of departing conspecifics, they also integrate information about the steady state of still non-departed conspecifics.

### Functional consequences

The evolutionary advantages of the distribution of departure latencies for species subject to predation are now well known [4], and survival typically increases with an increasing group size [20]. The individual benefit of being in a group is therefore often an increasing function of the number of individuals *N*, at least up to some maximal extent [4]. Considering two populations, staying (*S*) and departed (*D*), the individual benefit can be estimated as:
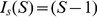
(3a)

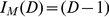
(3b)


The individual benefit 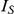 (equation 3a) and *I_M_* (equation 3b) are assumed to be proportional to the number of individuals being in the same behavioural state as the focal individual. When the *i* ^th^ individual departs, *i* is the rank of its departure and the transition for the whole group is:
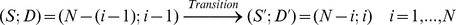
so that its benefit Δ*I* of staying compared to moving is the difference between the benefits to join the departing group and the benefits to remain with staying individuals expressed as:
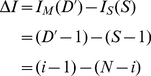
(4a)


Equation 4*a* allows the calculation of Δ*I*, and model 3 allows us to predict departure rates in group sizes untested in our experiments.

**Figure 4:**
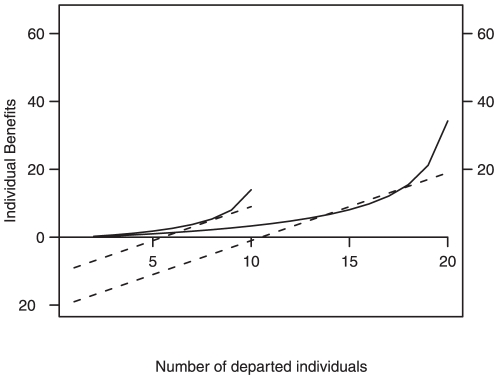
Scalability of the sheep decision-making process. Benefit difference ΔI (dotted) when switching from staying and moving, and departure rates μ (plain) as function of departure rank for groups of 10 and 20 individuals. Departure rates here, follow 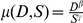 (model 3 with α = 1). Note that the benefits are proportional to group size.

Following a generic anti-predator strategy, it is beneficial to remain with the largest population. For a group of fixed size *N*, it is advantageous to remain while the number of departed individuals *D*<*N*/2, whereas it becomes beneficial to depart when *D*>*N*/2, and the benefit becomes positive (Δ*I*>0) when the departure rank *i*>*N*/2. Figure 4 demonstrates that the departure rates follow the same pattern for any group sizes. In fact, the experimental departure rate also increases with an increasing number of departed individuals (Fig. 2A), and increases sharply when the number of departed individuals is greater than half of the group, whatever the group size (Fig. 4). An important property is therefore that the sheep decision-making process can scale and function effectively, for any group size.

**Figure 5:**
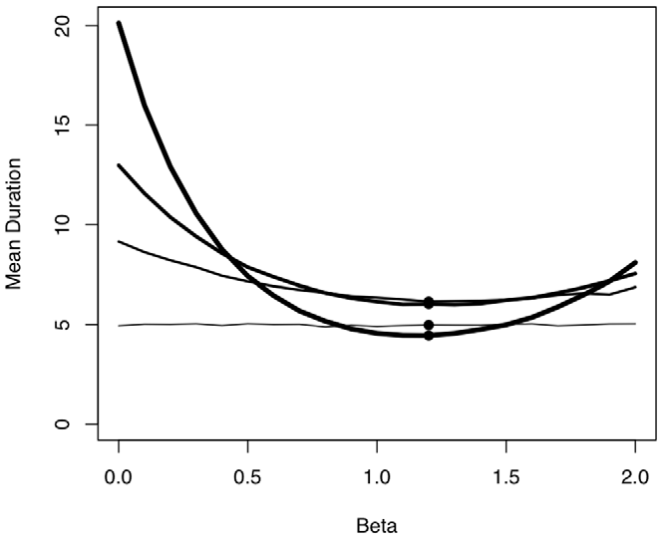
Sensitivity of the collective dynamics to the parameter β. Mean duration of the collective move for different group size *N* = 2, 4, 8, 32 (from thin curve to thick curve respectively), as a function of different values of β. The black dots represent the minimum value of mean duration for each group size.

How sensitive are the collective dynamics to the parameters we found? In our experiments we found that the balanced effects of departed and non-departed individuals follow β = 2*γ, with β = 1.2. Using equation 2, we tested the sensitivity of the mean duration of collective moves (the latency from the initiator departure to the last follower's departure) to deviations of β (0<β<2, γ = β/2, for different group sizes *N* = 2, 4, 8 and 32, Fig. 5). The mean duration was found to depend strongly on the interaction of *β* and group size. The values of β and γ for which the mean duration was minimized were similar to those found in our experiment, and furthermore they maintained this property of minimizing the duration of split events whatever the group size considered. In addition, the variation of mean duration for all group sizes is also minimized for the values that best fit our experiments. This is likely to have an important functional consequence to group decision making, because it facilitates consensus, functions independently of group size and minimizes the proportion of time the group is split during the act of decision-making.

## Discussion

By analyzing the dynamics of individuals' reactions within an experimentally-induced collective departure under controlled conditions, we have been able to demonstrate that individual decision-making in sheep is based both on departed and non-departed group members. This mechanism is scalable in four group sizes of eight or less individuals, and the experimental parameters' values β and γ proved to be the ones which minimize the duration of fission events, whatever the group size.

The temporal organization of individuals' responses was used to reveal the underlying individual decision-making process. This direct measurement of the individual sheep response demonstrated that their probability per unit time to display a response is constant over time as long as the stimulus remains the same (thus it is Markovian), and that both departed and non-departed individuals were *necessary* to account for these responses. The fit of the data at the collective scale showed that they were also *sufficient*. In other words, this is a parsimonious model that still fully explains the experimental results. We advocate that such a precise measurement should be made at both scales to fully explain collective behaviours.

Ward *et al.* [9] and Sumpter *et al.* [16] previously proposed a model to account for moving decision in a Y-maze in three-spine sticklebacks, *Gasterosteus aculeatus*. They suggested that individuals' probability of following leaders increases sharply with an increasing group size of departing individuals, and that following behaviour is inhibited by undecided (non-departed) individuals. However, they fit their model only to the collective response, and thus the individual parameters remained hypothetical. The present study gives further support to their hypotheses that both departed and non-departed individuals matter. Petit *et al.* [21] recently proposed a model for the collective decision in one group of white-faced capuchin monkeys *Cebus capucinus*. The movement initiator was highly prone to cancel the movement when too few group members followed in a too short time. In their model, the probability following was also modulated by the ratio of the departed to the non-departed group members, and it appears to be a quorum at the collective level. However, disentangling both effects still has to be validated using different group sizes.

Using different group sizes and individual measures which largely derive from a quantification of the dynamics at the collective scale in Merino sheep, Gautrais *et al*. [22] developed a model explaining the synchronization of states (resting and grazing) among group members. The transition between the two activities was also influenced both by active and inactive neighbours. Unlike this study, here we quantify the behaviour at the individual scale, and moreover, we control the initiation of the phenomenon. This methodology allows to isolate and highlight the social interactions and to exclude the contribution of any external stimulus.

Our experimental model suggests that individual decisions to follow those that have departed is not a quorum-like response because the probability monotonically increases with the ratio of already departed and non-departed individuals. As a consequence, the probability of response to the number of departed is dependent on group-size. Moreover, all individuals would instantaneously follow the trained individual which moved away. Finally, it engaged all potential followers every time, and so there is no apparent threshold under which no moving at all would occur, as was found in ants and fish [9], [11].

It is also noteworthy that the mean departure latency of the whole group did not vary with the experimental group size. This result stems from two balancing effects: the mean latency of the first follower decreases with group size (Fig. 3H), while the mean duration (from the trained individuals' departure to the last follower's departures) increases with it (Fig. 3I). The mean latency of the first follower decreases proportionally with (≈*N* ^−0,5^). This results from coupling between a pure sampling effect proportional to the number of potential followers *N* (see Appendix S2), and the individual departure rate decreasing by √*N*. One prediction of the inhibiting effect of the non-departed individuals is that large groups should be more stable because their members should be less sensitive to initiations. To explain the formation of large animal groups, the first effect may therefore be necessary to over-compensate the latter.

Natural selection is likely to result in decision-making rules that allow individuals to vary their behaviour efficiently across a wide range of environmental and social conditions [23], [24]. Our results in sheep are congruent with this concept. First, the decision-making rule is scalable. Secondly, the experimentally estimated parameters β and γminimized the time needed for all group members to depart. In fact, we found that the parameter values that minimize the duration of group splitting correspond to the experimental ones. Sheep responses were compatible with a strategy that minimizes putative risks of predation by choosing to stay with the larger group (Fig. 5) whatever the size of the departed and non-departed groups (Figs. 4 and 5): low departure rate for the (*N*/2) first movers (long departure latency = 1/departure rate, see Data analysis and Appendix S1), and when *N*/2 individuals have moved, they move as a cohesive unit with an increasing departure rate (Fig. 2). Following these arguments, the first mover may still pay disproportionate costs when departing from the group. If the first mover possesses knowledge of the environment with respect to its most profitable foraging areas, the benefit of moving to a new food resource could compensate the cost of the predation risk. Social foragers are known to have to make a compromise between food and safety [4], [25]. In our experiment the first mover is a trained individual which possesses pertinent information (the location of food reward) giving it a foraging advantage. Following social foraging theory, we can assume that for such individuals the benefits likely outweigh the risks incurred from moving away from the group, when the new area is not far (range: 10–20 m). This assumption could be tested in future experiments, for example by varying the reward given to the trained individuals, or the distance over which they must move.

Since we used relatively small group sizes, we assumed that each individual was able to monitor continuously the behavioural state of each member of the group, but this assumption would become irrelevant in large groups where crowding restricts perception of others that are beyond immediate neighbours. In any case, even in large groups, one should consider only the closest neighbours as stimuli (*sensu* Voronoi neighbours [26]). Hence the sensitivity both to departed and non-departed neighbours should stand robustly also in large groups, but perhaps with different parameter values (α, β, γ) [27]. Finally, to investigate the full dynamics of collective motion in large groups [26], [28], [29], models of individual reactions to neighbours inspired by the principles we found in the present study are likely to be informative. One would additionally need to quantify spontaneous initiations and the propagation of its effect across the group in order to understand how the following by the nearest neighbours would in turn trigger or not the departure of the farthest individuals [30].

Considering the ubiquitous nature of gregariousness observed in grazing herbivores, we therefore expect that in a wide range of ungulate species and more generally in many vertebrates, individuals may follow rules that conform to the general principles revealed here.

Moreover, we assume that similar individual rules could be at work in situations where group members are confronted with different alternatives like activities or directions, particularly in the presence of trained individuals [8], [31].

## Material and Methods

### Experimental set-up

Fieldwork was carried out in the experimental farm of Domaine du Merle (5.74°E, 48.50°N) in the South of France from January 2008 to March 2008, with females of Merinos d'Arles (three years old). The training set and the naïve set comprised 25 and 150 ewes respectively, which were randomly selected from a flock of 1600 females avoiding relatedness. Each ewe was marked on its back using a special paint in order to be identified. All the ewes were released every morning into enclosed paddocks situated within homogeneous meadows of Crau hay. The naïve set was penned up each evening in the same sheepfold as the training set.

To investigate the dynamics of decision making, we triggered movement using an informed individual [23]. This series of experiments was realized with the same training and experiments procedures as in our previous study [23]. Sheep were trained (in five groups of five sheep) to become movement initiators. After two weeks of training, we obtained four trained, one well trained individual per training group, which answer on 95% of the test. Then, one trained individual was combined with sheep familiar with the sound and the panel, but naïve for the food target, i.e. habituated to the stimulus, and we used different group sizes: groups consisted of two (*N* = 11 replications), four (*N* = 7), six (*N* = 11) and eight individuals (*N* = 11), to obtain different arrangements of the number of departed *D* and non-departed *S* individuals. Groups of sheep were introduced in circular arenas (25 m diameter), in a flat homogeneous pasture [23]. Arenas were enclosed with sheep fences and visually isolated from immediate surrounding by a green polypropylene net. In each group tested, one trained sheep initiated a move towards a coloured panel raised under experimenter's control. Under these controlled condition, individual decisions to move depended mainly on other group members' behaviour. For that purpose a food reward was delivered on the ground at the foot of one of five panels laid at the periphery of the arena. Before raising one panel, a sound stimulus was delivered to synchronize the attention state of all sheep (head-up) so they could concurrently perceive the departure of the initiator. This could be compared to a situation of heightened attention of all group members such as may occur under conditions of predation risk in which it is important to be vigilant and to flee if necessary.

The behaviour of sheep was recorded with a digital camera (Canon EOS D50) fixed on the top of the tower with the frequency of 1 frame per second.

We use several trained individuals in order to prevent collective movements in response to behaviour of one potentially peculiar initiator. All naïve individuals were tested only once. We triggered a departure of only one individual in each group.

Animal care and experimental manipulations were in accordance with the rules of the French Ethical Committee for animal experimentation.

### Data analysis

The behaviour of each individual was quantified using a probabilistic stimulus/response function. Latency of the follower *i* corresponds to the time elapsed (in seconds) since the previous departure of individual *i*−1. The distributions of experimental following latencies fitted exponential distributions, indicating that the probability per unit time to depart (the log gradient of the exponential distribution) is constant over time for the same herd configuration (number of departed and non-departed). The experimental probability per unit time to follow (the following rate expressed in s^−1^) is the inverse of the mean departure latency. The latencies were gathered as a function of the number of *D* (departed) and the number of *S* (non-departed) individuals (see also Appendix S1). Most departures were well-defined and discrete events in our time scale, but when we observed two or three individuals departing simultaneously (within the same second), they are ascribed to the same departure rank and thus submitted to the same combination of number of departed and non-departed individuals.

To perform our analysis at the individual scale, we assumed that the individual response functions were the same for all naïve individuals, and were stable over time. This is reasonable since naïve individuals were used only once, so that any potential effects of learning, exploration, habituation or uncontrolled social experience were discarded. This precluded also any potential effect of inter-individual affinity [8]. Moreover, the trained sheep had the same motivation to depart towards the panel and exhibited the same movement away from the group, that is the trained sheep walked directly to it [23]. No naïve sheep departed towards the target before the trained sheep [23], and when they departed, they move towards the trained sheep (and not the panel). In control groups, with no trained sheep, naïve individuals never walked towards the panel [23]. This strongly suggests that naïves were engaged in a pure following behaviour.

### Response function fitting

A simple linear regression on the log-transformed data was used to fit the parameters of the response function.

## Supporting Information

Appendix S1 Equation of followers' departure time.
(0.08 MB DOC)

Appendix S2 Individual and collective quantification of the probability of following. In the simplest case, individuals respond independently to the stimulus (onset at time T0), with the same intensity. Since the response is an event (a departure), the response intensity is reflected by its latency coming out from the individual probability per unit time displaying the response event and the number of individuals. This individual probability per unit time displaying the response (in this study, following) is constant over time as long as the stimulus remains the same, and jumps to a new value when the stimulus changes. The figure S1 sums up the situation for a group of three individuals, which respond independently to a common stimulus (one group members departure) with a common and constant probability R. The stimulus onset (the trained departure) is at time T0 and remains the same until the departure of one conspecifics, individual a in our case, displays the response at time T1. Individual b displays the response at time T2, individual c at time T3 and individual d at time T4. On the left, the individual departure time is represented separately for each individual (Indiv a to d). At time T0, the probability per unit time to observe the departure of the first follower jumps from 0 (before the stimulus) to the probability to follow Pa after T0 and becomes irrelevant as soon as they have displayed the response. On the right, the corresponding probability seeing one departure per unit time is represented. Between T0 and T1, four individuals are liable to depart, hence the probability seeing one of them to do it is four times the individual rate. Between T1 and T2, only three individuals are now liable to display the response, hence the probability seeing one of them to do it falls down to three times the individual rate, and so on… Correspondingly, the experimental probability per unit time seeing one departure has to be corrected by the number of individuals liable to depart. So, to recover the individual departure rates, statistics of departure were gathered separately for each set of situations comprising the same number of individuals: one set where all the individuals were still present (from T0 to the first departure), one set where every individual but one were still present (from the first departure to the second one), etc. The individual departure rates were then obtained by dividing the corresponding mean departure rate by the number of individuals that were present. This procedure remains the same even when the departure rate depends on the number of the individuals that have already departed.
(1.56 MB TIF)
