# Supplementary material for: Scalable Rules for Coherent Group Motion in a Gregarious Vertebrate
Source: PLoS One. 2011 Jan 5;6(1):e14487. doi: 10.1371/journal.pone.0014487 (PMC3016320; doi:10.1371/journal.pone.0014487)
Supplement: Appendix S1 — Equation of followers' departure time. (0.08 MB DOC) [file pone.0014487.s001.doc]

**Appendix S1. Equation of followers’ departure time.**

The master equation, a set of first-order differential equations, describes the time evolution of the probability of the system (*P_i_*) to occupy each one of the discrete set of states. *P_i_* is the probability for the system to be in the state *i* where *i* individuals are following the initiator (*i = 0, …, N*), the size of the group being = *N+1*. The time evolution of is given in terms of a birth and death processes. For each equation , we have a birth term corresponding to the departure of the *i*^th^ follower:

(B1a)

and a death term corresponding to the departure of the *i*+1^th^ follower

. (B1)

*P_i-1_* (*P_i_*) is the probability of being in the state *i-1* (*i*) followers and *k_i-1_* (*k_i_*) is the transition probability corresponding to the departure of the *i*^th^ (*i+1*^th^) follower. Thus

. (B2a)

There are two exceptions for *P_0_* with only a death term and for *P_N_* with only a birth term

(B2b)

(B2c)

The initial conditions at *t* = 0 are *P_0_* = 1, *P_i_* = 0 for *i* > 0.

*k_i_*, the transition probability between *i* and *i* +1, is equal to the individual probability per unit of time of moving $m_{i}$ multiplied by *N* – *i*, the number of potential followers still at rest (*i.e.* non-departed: see Appendix A):

. (B3a)

The general shape of $m_{i}$ is

(B3)

where *i* + 1 and *N* - *i* are respectively the number of departed (the sum of the number of followers and the initiator) and non-departed individuals (see equation 1, line 131) .

α imposes the time-scale of the dynamics.

The solutions are:

(B4a)

with

(B4b)

(B4c)

(B4d)

The mean departure time of the *i*^th^ follower and its variance are:

(B5a)

(B5b)
