# Supplementary figures and images for: Scalable Rules for Coherent Group Motion in a Gregarious Vertebrate
Source: PLoS One. 2011 Jan 5;6(1):e14487. doi: 10.1371/journal.pone.0014487 (PMC3016320; doi:10.1371/journal.pone.0014487)

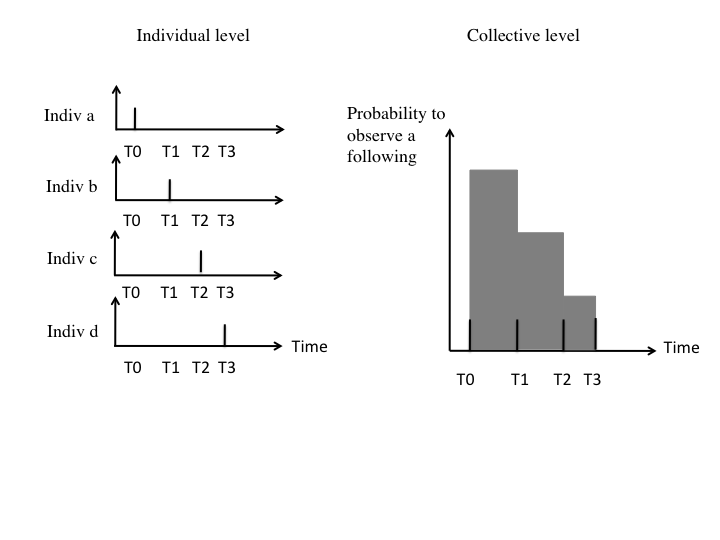

Supplement: Appendix S2 — Individual and collective quantification of the probability of following. In the simplest case, individuals respond independently to the stimulus (onset at time T0), with the same intensity. Since the response is an event (a departure), the response intensity is reflected by its latency coming out from the individual probability per unit time displaying the response event and the number of individuals. This individual probability per unit time displaying the response (in this study, following) is constant over time as long as the stimulus remains the same, and jumps to a new value when the stimulus changes. The figure S1 sums up the situation for a group of three individuals, which respond independently to a common stimulus (one group members departure) with a common and constant probability R. The stimulus onset (the trained departure) is at time T0 and remains the same until the departure of one conspecifics, individual a in our case, displays the response at time T1. Individual b displays the response at time T2, individual c at time T3 and individual d at time T4. On the left, the individual departure time is represented separately for each individual (Indiv a to d). At time T0, the probability per unit time to observe the departure of the first follower jumps from 0 (before the stimulus) to the probability to follow Pa after T0 and becomes irrelevant as soon as they have displayed the response. On the right, the corresponding probability seeing one departure per unit time is represented. Between T0 and T1, four individuals are liable to depart, hence the probability seeing one of them to do it is four times the individual rate. Between T1 and T2, only three individuals are now liable to display the response, hence the probability seeing one of them to do it falls down to three times the individual rate, and so on… Correspondingly, the experimental probability per unit time seeing one departure has to be corrected by the number of individuals liable to depart. S [file pone.0014487.s002.tif]
